# What do we tell patients about elective total hip replacement in the UK? An analysis of patient literature

**DOI:** 10.1186/1471-2474-14-152

**Published:** 2013-05-01

**Authors:** Avril Drummond, Claire Edwards, Carol Coole, Catherine Brewin

**Affiliations:** 1 Faculty of Medicine and Health Sciences, University of Nottingham, A Floor, South link corridor, Queen’s Medical Centre (QMC), Nottingham, NG7 2UH, UK; 2 Faculty of Medicine and Health Sciences, University of Nottingham, Queen’s Medical Centre, Nottingham, UK; 3 Senior Occupational Therapist in Orthopaedics, Nottingham University Hospitals NHS Trust, Hucknall Road, Nottingham, UK

**Keywords:** Total hip replacement, Hip precautions, Hip restrictions, Patient information, Occupational therapy

## Abstract

**Background:**

Although hip information literature is given to people following total hip replacement (THR) almost routinely, little evaluation has been conducted on it to date. Our aim was therefore to analyse and evaluate the literature provided to patients by occupational therapists concerning elective hip surgery in the UK.

**Methods:**

This was a pragmatic, descriptive analysis of information leaflets routinely given to patients undergoing primary total hip replacement (THR). The literature was collected as part of a national survey of occupational therapy practice. In the absence of a suitable evaluation tool, the patient leaflets were compared using a checklist devised by the researchers. The three areas of interest were: accessibility including presentation of information, breadth of information covered and specific activities of daily living described.

**Results:**

111 information leaflets and booklets were examined. These ranged from hospital publications which were professionally printed to those produced by individual departments. There was a variation in the readability of the leaflets ranging from 13% to 83%; the mean was 45% (SD 15). There was also variation in the content ranging from those covering surgery and possible complications, to those including diet and hip exercises. The most commonly covered activity of daily living was advice on sitting (99; 89%); the least commonly covered was work (26; 23%). Only 3 (2.7%) booklets had involved patients in their production and only 22 (20%) signposted obtaining information in another language or in Braille.

**Conclusions:**

There was a range of literature in terms of presentation and content given to people who had a total hip replacement (THR). Although some booklets and leaflets scored highly, some did not meet basic standards such as providing contact details for help, using good quality diagrams, suggesting further reading or involving patients in their design. These results highlight important and fundamental deficiencies in the literature routinely provided.

## Background

Elective total hip replacement is increasingly common with more than 70,000 operations performed in England and Wales between 2010 and 2011, compared with 56,000 in 2005 [1]. To reduce the risk of hip dislocation following surgery, staff routinely advise patients to follow certain post-operative restrictions commonly known as hip precautions [2]. These precautions usually involve advising patients not to flex their hip beyond 90% and not to adduct, medially or laterally rotate the hip joint. Such restrictions in movement have direct implications for everyday activities, ranging from using the toilet to driving. Yet although occupational therapists routinely apply hip precautions and provide equipment, national practice differs in both delivery and management [3,4]. However it would seem that the majority of occupational therapists commonly provide written information to patients undergoing total hip replacement [4].

Written information is a key means of informing, educating and involving patients [5] and enhances communication between healthcare professionals and patients. Providing good patient information is recognised as an important part of enhanced or rapid recovery programmes [6]. However leaflets may not be targeted appropriately and, for example, there may be a discrepancy between reading ability and the written information presented in the patient education leaflets [7]. There can also be concerns such as whether the aim of the literature is clear [8].

To date there has been relatively little evaluation of the literature given to people undergoing orthopaedic surgery or hip surgery. Evaluations have tended to examine literature only as part of a package of care [9,10]. Johansson et al. [11] conducted an evaluation of 25 educational orthopaedic materials provided by one hospital. This ranged across all orthopaedic care and included material covering upper and lower limb, plaster casts and aids. The authors reported that although information was largely presented well, there were concerns about some content; some were out of date and the overall aim of some was not obvious.

Although it would seem that patient information leaflets are given to people following elective THR routinely [4], little previous research has studied the quality or value of this information. This is of particular interest in view of the increasing number of operations, and the overall reduction in length of stay. Length of stay for primary hip replacement has reduced in recent years with an average UK length of stay of 11 days during 1998–99 [12], compared with 8 days in 2002 [13]. Shorter stay would appear to reduce the time available for clinical staff to address patients’ knowledge and understanding and thus the adequacy of written information may be important. Therefore the aim of this study was to evaluate hip precaution literature given to people undergoing a THR by occupational therapists.

## Methods

We confirmed that no ethics or R&D permissions were needed from our University Ethics office who regarded this study as a service evaluation. Patient information leaflets were collected as part of a national survey of occupational therapy hip precautions following elective total hip replacement. The details of this study have been published elsewhere [4] however, in summary, 263 questionnaires were posted to UK occupational therapists to identify their current day to day practice in primary THR. Of the responses, 174 (66%) questionnaires were analysed. 170 (97.7%) occupational therapists said they supplied their patients with written information and 121 (70%) enclosed copies of the leaflets they routinely gave to patients pre and/or post THR.

There were three broad areas we were interested in. Firstly we wanted to evaluate the accessibility of the information in terms of how it was presented to a lay audience. Secondly we wanted to establish if the leaflet was solely an occupational therapy publication or if its scope was wider. If the remit was wider, we wished to examine what other areas were covered. Finally we wanted to establish whether specific activities of daily living (ADLs) were presented and if so, what these were.

In deciding how to evaluate the leaflets the authors considered a number of existing tools for evaluating patient literature; we were particularly interested in using tools which had involved patients in their development. Thus we included the International Patient Decision Aid Standards (IPDAS) [14], DISCERN handbook [15], Ensuring Quality Information for Patients (EQIP) [16] and the British Medical Association patient information appraisal system [17]. However many of the criteria described in these tools were not directly applicable for hip leaflets. For example several concerned criteria such as the balance or bias of the information [14,17], the choice of treatment options available [15] or benefits [16] of different treatments. Some criteria were valuable and had potential to be used, such as: plain English [14]; appropriate typeface [17]; appropriate length [17]; contained the name of the person/ department who produced it [16]; whether patients were involved in its production [16]; details of additional sources of support and information [15].

The authors discussed and agreed the factors they believed important in producing a guide for patients. They used these to develop a bespoke evaluation instrument based on questions from the existing tools and from their clinical knowledge. The three areas were converted into three sections of the tool:

1. Accessibility of information for patients. This was determined by a combination of factors including: structure of the material presented and whether it was logical; size of font, use of lay language and lack of jargon; the use of diagrams/photographs to illustrate points; whether patients had been involved in the preparation of the guide.

2. General content. We examined whether the document was multi-disciplinary based or profession specific. Thus we examined if particular aspects of care were covered such as details of the surgery, wound care, complications of surgery , specific exercises, diet, and general hip precautions, e.g. do not bend more than 90 degrees when sitting down, do not cross your legs.

3. As these leaflets were distributed by occupational therapists, we wanted to establish if specific ADLs were covered. The activities selected were based on the results from the survey we had conducted previously [4].

The three sections of the evaluation tool were scored separately. The first section was scored as either ‘no’ (0 point), ‘partly’ (1 point) or ‘yes’ (2 points). The second and third could only be scored either ‘yes’ or ‘no’.

After agreeing the content of the questionnaire and the method of scoring, initial usability of the evaluation tool was checked independently. Three researchers scored a random sample of 30 leaflets to establish the content validity by ensuring each item contributed to the assessment of the document. To agree on scoring, researchers discussed differences in judgement on the appropriate size of documents, the usefulness of diagrams and how to define the criteria for scoring referenced statements and contact details for help. Additional notes and examples were added to the checklist to ensure greater reliability. Several clarifications were noted as a result. For example, illustrations had to be good quality and patient focused; supplying the hospital phone number was not appropriate as a contact for further information.

The researchers also agreed to suspend clinical opinions when marking the documents and to assess on the readability of the leaflet alone, not whether they judged the information to be correct. Thus if material was provided on diet - whether the advice was considered good or poor - that category would be ticked.

However if an item was not actually presented - for example if the entry said only that the patient would be shown how to do an activity by the occupational therapist (i.e. no actual information provided), this would not be regarded as informative. For example, some included ‘dressing’ but underneath the heading stated that this would be demonstrated in hospital. Some categories were re-defined as a result of the pilot testing, for example ‘travelling as a passenger in a car’ and ‘driving’ were added separately, and ‘mobility’ was divided into ‘walking’ and ‘climbing stairs’. We also added the category ‘picking something off the floor’ as our initial impression was that several leaflets referred to this.

After the pilot phase, all documents were assessed by one researcher and then independently by a second researcher. This included re-scoring the 30 leaflets used in the pilot exercise. At several time points the researchers met to discuss their scores and specifically discrepancies in scores. We judged each document separately, although accepted that a number were intended to be issued in conjunction with other leaflets. However we felt each leaflet should be able to stand alone if a supporting document had not been provided or was lost.

## Results

121 leaflets or information booklets routinely given to patients pre and/or post THR were examined. Of these, ten were excluded from this study: nine were duplicates and one concerned the general availability of orthopaedic services. Ten further leaflets were noted to be paired with one or more other documents (a total of 21 leaflets).

### Accessibility of literature

There was a wide range of material submitted which ranged from glossy, professionally produced booklets to a single sheet of paper. Some of the publications were original copies while others were photocopies. Some leaflets were produced by an occupational therapy department, some from an orthopaedic directorate and others by the hospital or Trust. The shortest publication was a single side of paper and the longest was a 50 page booklet. The overall scores on presentation ranged from 13% to 83% (mean 45.16%; SD 14.93); the scores awarded for individual items may be seen in Table 1.

**Table 1:** Accessibility of hip leaflets

**Accessibility of leaflet**	**n (%)**					
	**No**		**Partly**		**Yes**	
**Name of person/ department provided**	41	(36.9)	27	(24.3)	43	(38.7)
**Appropriate size**	24	(21.6)	24	(21.6)	63	(56.8)
**Well laid out**	8	(7.2)	35	(31.5)	68	(61.3)
**Patients/ families involved in production**	106	(95.5)	2	(1.8)	3	(2.7)
**Appropriate typeface**	2	(1.8)	15	(13.5)	94	(84.7)
**Illustration/ diagrams included**	29	(26.1)	45	(40.5)	37	(33.3)
**Everyday language used**	1	(0.9)	29	(26.1)	81	(73.0)
**Case studies / patient anecdotes included**	111	(100.0)	0	(0.0)	0	(0.0)
**Contact details provided**	36	(32.4)	27	(24.5)	48	(43.2)
**Information for non-English speakers**	86	(77.5)	3	(2.7)	22	(19.8)
**Space for recording personal information**	82	(73.9)	10	(9.0)	19	(17.1)
**Referenced statements/ further reading**	92	(82.9)	8	(7.2)	11	(9.9)

The highest total score for a leaflet was 83% followed by 71%. These two leaflets were characterised by clear text, the inclusion of many useful diagrams to support the text, headings, logical order, contact details for help, guidance for obtaining copies in another language or Braille. One was twenty pages with a contents page and space for own notes; the other was 24 pages long. Both had a clear title referring to caring for the hip after surgery.

By comparison the lowest total score was 13% (obtained by two leaflets). These were one and two pages long respectively and had one poor quality diagram each. One had handwritten text inserted on the final copy - which was difficult to read. The title of one was ‘Precautions’ and it was not initially clear that this referred to hip precautions.

64 (57.7%) leaflets included their ‘produced’ or ‘revised’ date. In 18 (28.1%) leaflets it was not clear if the date given was when a review of the material was planned or if this was the date the review had actually been completed. One leaflet was a final draft about to go to print. More than 27 leaflets (24%) were more than two years old; eight (7%) were more than five years old and, of these, two (2%) were more than nine years old.

With regard to contact details for patients who had a query about their care, some leaflets only listed a main hospital telephone number. This was only scored as ‘partly’ as, even though it was regarded as better than no details, it was not thought very informative. Therefore only 48 (43%) met this criteria.

We also found that few publications made reference to signposting people who needed information in another language or in Braille. It is possible that this reflected the needs of the local population served, for example, whether there was a large ethnic population using the service. However only 22 (20%) gave information on access to the material in Braille or other language.

### Breadth of information

The leaflets we examined were those issued by occupational therapists. For the majority, details were not clearly given on who had written the guide - either a person, a professional or a department; 27 (24%) ‘partly’ did and 41 (37%) did not. However some were very clearly written by occupational therapists to cover principally occupational therapy aspects of care. We classified 54 (48.6%) as ‘mainly’ an occupational therapy publication. However some were more rehabilitation focused (e.g. occupational therapy and physiotherapy) and some were from the whole orthopaedic team (covering surgery and complications of surgery). We found that 49 (44.1%) included material relating to describing the actual operation, 39 (35.1%) provided advice on pre-operative care and 40 (36%) covered information on medical aspects such as pain relief and possible complications such as DVT and wound infection. Forty-six (41.4%) provided advice on general health issues such as keeping fit before and after surgery, and diet and nutritional recommendations, while 44 (39.6%) included specific exercises for immediately before or after surgery or after discharge from hospital. Generic advice on hip precautions - such as not bending more than ninety degrees or crossing legs in sitting - was given by 109 (98.2%).

### Specific activities of daily living

Table 2 details the ADL items covered by the leaflets. No leaflet covered all of the activities listed. The mean number of activities covered by each booklet was 9.77 (SD 4.55); the range was from 0–17 activities. The most commonly covered activity was advice on sitting (99; 89%) and the least was work (26; 23%). Under ‘other’, ADL specific activities were listed such as advice on flying (8; 7.2%), looking after pets (3; 2.7%), using public transport (3; 2.7%) and getting up after a fall (1; 0.9%).

**Table 2:** Activities of daily living covered

**Activities**	**n (%)**			
	**No**		**Yes**	
**Sitting**	12	(10.8)	99	(89.2)
**Bathing**	24	(21.6)	87	(78.4)
**Driving a car**	26	(23.4)	85	(76.6)
**Sleeping**	27	(24.3)	84	(75.7)
**Dressing/ undressing**	29	(26.1)	82	(73.9)
**Being a (car) passenger**	29	(26.1)	82	(73.9)
**Using the toilet**	39	(35.1)	72	(64.9)
**General domestic activities**	41	(36.9)	70	(63.1)
**Modifying the home environment**	47	(42.3)	64	(57.7)
**Sex**	49	(44.1)	62	(55.9)
**Bed transfers**	51	(45.9)	60	(54.1)
**Stairs**	62	(55.9)	49	(44.1)
**Leisure**	65	(58.6)	46	(41.4)
**Walking**	66	(59.5)	45	(40.5)
**Social support**	69	(62.2)	42	(37.8)
**Bending down/ picking something up from the floor**	81	(73.0)	30	(27.0)
**Work**	85	(76.6)	26	(23.4)
Other*	96	(86.5)	15	(13.5)

* E.g. flying, looking after pets, using public transport, getting up after a fall.

## Discussion

We found that the hip precaution literature varied greatly in terms of both presentation and content. This was in keeping with the observations of Johansson et al. [11] and suggests that more attention needs to be paid to the design and substance of information leaflets for patients.

There were several important limitations to our results. Firstly, in the absence of a suitable tool, we had to devise the evaluation instrument ourselves. Although this was based on other relevant literature [14-17] and was well tested within our team, it would have been preferable to use an existing tool. We did not formally test the psychometric properties of the instrument, as this was not the focus of this research, but recognise this was a limitation. There were also some obvious difficulties with our tool; we decided not to evaluate the quality of the actual advice given and concentrated on whether or not a topic was discussed. Thus we might have given a high score to a leaflet when experts might have felt the advice given was inappropriate.

There are also problems with regard to the scope of the literature we examined. Some leaflets were meant to be used in conjunction with other leaflets. However we took a pragmatic decision that patients could mislay parts of a package of literature and therefore examined each individually; it seemed that at least two of the leaflets we received should have had another one with them, but had not been sent - validating our decision to examine each individually. Conversely some may feel this was unfair as individual leaflets were taken out of context and consequently did not cover all the aspects of care we evaluated, for example, if there was a separate leaflet issued from physiotherapy, mobility and stairs may not have been covered; equally complications after surgery could have been covered elsewhere.

We did not ask at what stage leaflets were given out. Some might have been given out weeks before surgery, some on the ward after surgery. Thus if someone had already had surgery, topics such as losing weight, reducing smoking and surgical details might not have been helpful. On the other hand, with the current emphasis on pre-operative assessment for elective hip surgery, it is likely that most leaflets are targeted at people prior to hospital admission.

Another potential criticism is that such leaflets are used as an adjunct to therapy and therefore should not be evaluated out of context. Indeed some believe that leaflets alone have little impact [5] and that written information should be combined with verbal information [18]. Although this point is well made, the fact remains that, because of reduced length of stay, patients have to take more responsibility for their own rehabilitation and consequently written literature is increasingly important in recovery.

Yet notwithstanding the study limitations, there were very interesting findings. Generally the literature was presented clearly and without jargon and the majority of leaflets had clear diagrams to accompany and illustrate the text. There seemed to be marked differences in the amount of more general information included (e.g. diet, complications, pain). Whilst this might depend on the overall aim of the leaflet there were other omissions which were more critical - for example who the patient should contact if there were problems after discharge, the date when the leaflet had been updated or written. There were great differences in the actual quality of the paper used and presentation in terms of whether leaflets were professionally printed or were photocopies which had started to fade over time. One of the most interesting observations made was that there was no evidence that patients had been consulted or involved in the production of any of the hip literature we reviewed. This raises the question of whether such literature should be formally reviewed by the patients who would use them, which would seem to be an obvious step forward.

Our findings suggest that health professionals producing literature for patients after THR should ensure that the aim, purpose and timing of leaflets are considered. Clinicians should not underestimate the time needed in the preparation of such material and, perhaps most importantly, patients should be involved in the production of this literature. Moreover, although our findings relate specifically to information leaflets given to patients following THR, many of the issues noted relating to the development, quality and evaluation of literature have been reported in a range of studies across a range of conditions [7,8,10,18,19]. Thus nationally and internationally there is growing evidence that literature for patients may not be fit for purpose; given the effects of this on recovery, anxiety and dependency, this must be a matter of concern.

## Conclusions

We found that that the hip precaution literature given to patients by their occupational therapists varied greatly in terms of presentation and in content. Although this study was conducted in the UK, and the survey was restricted, these results highlight important and fundamental deficiencies in the literature provided to patients. This has implications for orthopaedic centres who conduct elective THRs and who provide literature. Much more attention needs to be paid to the design and substance of information leaflets, most notably the inclusion of patients in the production of this material.

## Competing interests

The authors declare they have no competing interests.

## Authors’ contributions

AD contributed to the conception and design of the protocol, assessed literature, performed analysis, interpreted data and drafted the final paper. CE contributed to the design, assessed literature, conducted data entry, analysis, interpreted data and commented on drafts of the paper. CC contributed to the design, assessed literature, interpreted data and commented on drafts of the paper. CB contributed to the design, interpreted data and commented on drafts of the paper. All authors read and approved the final manuscript.

## Pre-publication history

The pre-publication history for this paper can be accessed here:

http://www.biomedcentral.com/1471-2474/14/152/prepub
